# Skin Gas-Based Two-Dimensional Framework for Assessing Stress-Related Symptom in Humans

**DOI:** 10.3390/s26216748

**Published:** 2026-10-25

**Authors:** Hideaki Yamaguchi, Kanae Maruyama, Eiji Fujioka, Akihiro Hosomi, Yoshika Sekine

**Affiliations:** 1 Advanced Development Department 1, AISIN CORPORATION, Kariya, Aichi, 448-8650, Japan; hide-y@body-t.aisin.co.jp (H.Y.);; 2 Course of Chemistry, Graduate School of Science, Tokai University, Hiratsuka City, Kanagawa, 259-1292, Japan

**Keywords:** skin gas, stress-related symptom, thermoregulation, passive flux sampler, blur sensation, non-invasive sensing, stress monitoring

## Abstract

**Highlights:**

**What are the main findings?**

- The findings from the larger dataset were consistent with those of our pilot study, supporting the use of integrated metabolic and vasomotor-related indices to visualize stress-related subjective states associated with “Blurriness” on a two-dimensional map.
- The distribution of “Blurriness” scores and seven-day measurements showed individual differences in stress-related states and their temporal trajectories.

**What are the implications of the main findings?**

- Skin gas profiling may provide a non-invasive approach for visualizing individual differences in stress-related subjective states.
- The proposed framework may help characterize temporal changes in individual stress-related states and support future development of personalized stress-monitoring approaches.

**Abstract:**

Stress is an important factor affecting human health and work performance, but non-invasive approaches for evaluating individual stress-related states remain limited. Human skin gases may provide physiological information related to stress and thermoregulation. In this study, we examined a skin gas-based framework for visualizing stress-related subjective states upon awakening. A total of 441 valid datasets were obtained from 100 participants. Subjective symptoms were assessed using the “Jikaku-sho Shirabe” questionnaire, which is widely used in Japan. Dermal emission fluxes of five skin gases (acetone, acetaldehyde, acetic acid, propionic acid, and hexanoic acid) were measured and converted into metabolic and vasomotor indices. Among the five questionnaire categories, the “Blurriness” score was significantly correlated with the metabolic activity index (*r* = 0.11, *p* < 0.05) and the vasomotor index (*r* = −0.17, *p* < 0.01). These indices were then plotted on a two-dimensional map, with the corresponding “Blurriness” scores assigned to each observation. The “Blurriness” scores were relatively low in the lower-right region and tended to increase toward the upper-left region, consistent with our previous pilot study. Seven-day measurements in six participants revealed individual differences in trajectories and cumulative movement across the map. These findings suggest that the proposed two-dimensional framework may provide a non-invasive approach for visualizing individual stress-related subjective states and their temporal changes. Further validation using objective measures and the development of sensors for rapid skin gas measurements may facilitate the practical application of this approach.

## 1. Introduction

Stress is a critical factor affecting human health and work performance, particularly in modern urban and occupational environments where workers are frequently exposed to psychological and physical demands [1,2]. Mental disorders among workers are now a leading cause of sickness absence and long-term work incapacity in many developed countries [3,4,5]. The majority of mental health problems observed in the workforce are related to common psychiatric conditions, such as depression, anxiety, and other stress-related conditions [3,6,7]. Questionnaire surveys are widely used to assess individual stress responses in occupational settings because of their simplicity and feasibility [8,9,10]. Such questionnaire-based assessments mainly reflect subjective perceptions of stress. However, discrepancies may occur between subjective assessments and physiological responses under chronic and/or high-stress conditions [11], and objective assessment using physiological indicators is considered necessary in addition to questionnaire-based assessments [12]. Measurements of physiological indicators, such as blood pressure, brain activity and heart rate variability, provide objective insights but often require complex instrumentation and may impose a burden on participants [13]. Biochemical markers, including blood cortisol and salivary amylase, offer reliable indicators of stress; however, blood sampling is invasive, and saliva collection requires strict hygiene control [14,15]. These limitations highlight the need for alternative, non-invasive sensing approaches suitable for monitoring in occupational settings.

Human skin gas is a complex mixture of inorganic and organic volatile compounds released from the skin surface and is often associated with body odor [16,17]. The composition of individual skin gas is known to respond to mental states such as nervousness and anxiety [18,19,20,21]. Asai et al. [19] demonstrated that dermal ammonia emission may be associated with heart rate variability indices related to autonomic nervous system activity, suggesting its potential as a non-invasive physiological monitoring approach. However, dermal ammonia monitoring has not yet been implemented in practical applications. In our previous pilot study [22], we proposed a novel framework for evaluating stress states based on skin gas emissions and questionnaire-based assessments. This framework was developed from the perspective of thermoregulation, an important function involved in maintaining human homeostasis. Because psychological stress can influence thermoregulatory processes, changes in thermoregulation may provide physiological information related to stress states [23,24].

Therefore, we focused on metabolic activity related to heat production and vasomotor activity related to heat dissipation and heat retention. We defined a metabolic activity index using acetone, acetaldehyde, and acetic acid, which are involved in metabolic pathways related to energy metabolism [25,26,27], and a vasomotor-related index using hexanoic acid and propionic acid, which have been associated with vascular regulation [28,29]. Based on these indices, we constructed a two-dimensional map, with the metabolic activity index on the vertical axis and the vasomotor-related index on the horizontal axis. The results showed a tendency for lower scores of perceived blur sensation in the subjective assessment to be distributed in the lower-right region of the map, whereas higher scores tended to be distributed toward the left side. We expected that this two-dimensional framework could enable the objective visualization of stress-related states through skin gas measurements and contribute to the prevention and mitigation of adverse health effects associated with work-related stress.

However, the pilot study was limited by its small sample size, comprising 12 participants and 72 data points. In the present study, we expanded the number of participants to 100 and analyzed 441 valid data points to increase the robustness of the previous findings. We compared the spatial distribution of skin gas emissions on the two-dimensional map with subjective stress-related symptoms assessed using a questionnaire. The aim of this study was to examine the potential of this skin gas-based sensing framework for the objective evaluation of stress-related physiological states.

## 2. Methods

### 2.1. Participants and Study Design

This study was conducted from November 2021 to February 2025 by recruiting a total of 100 healthy subjects consisting of 57 male (age: 42 ± 12) and 43 female (age: 37 ± 8.7) who were working for companies and industry associations. Individuals diagnosed with depression or other mental disorders by a physician were excluded from the study.

At the participants’ homes, human skin gas samples were collected within 30 min after waking up in the morning and for 1 h using a passive flux sampler (PFS) designed for volatile organic compounds (VOCs) [17,30,31]. During skin gas sampling, subjective fatigue was assessed using the questionnaire “Jikaku-sho Shirabe” [32,33,34]. During the 1-h sampling period, participants were instructed to remain seated and at rest, although light activities such as reading and computer work were permitted. However, they were instructed to minimize movement of the non-dominant hand, to which the sampler was attached. During the sampling period, only water or tea was permitted for consumption. After the skin gas sampling was initiated, participants joined an individually scheduled online meeting. During the video call, the researcher checked the attachment of the sampler and asked about the participant’s physical condition. After completion of sampling, no restrictions were placed on the participants’ subsequent activities, and they were instructed to continue their daily lives as usual.

The participants conducted the measurements for five consecutive working days, from Monday to Friday. Additionally, to investigate daily changes in subjective stress-related symptoms, including those during weekends and holidays, six participants conducted measurements for seven consecutive days, from Saturday to Friday.

### 2.2. Subjective Fatigue Assessment

To assess subjective fatigue among the participants, we used the questionnaire for work-related fatigue feelings, “Jikaku-sho Shirabe” [32,33,34], which was developed by the Industrial Fatigue Research Committee of the Japan Association of Industrial Health in 2002. This questionnaire is widely used in Japan to assess subjective fatigue among workers. The questionnaire consists of 25 items, and participants were asked to rate each item on a five-point Likert-type scale ranging from “not applicable at all” (score = 1) to “very applicable” (score = 5). The 25 items are grouped into five categories corresponding to “Sleepiness” (feeling like yawning, drowsiness, lack of motivation, tiredness throughout the body, and a desire to lie down), “Instability” (feeling nervous, restless, anxious, depressed, and having difficulty thinking), “Discomfort” (a heavy feeling in the head, feeling ill, headache, feeling muddled or mentally confused, and dizziness), “Sluggishness” (stiffness in the neck and shoulders, pain in the hands and fingers, dullness in the arms, lower back pain, and tiredness in the legs), and “Blurriness” (dry eyes, eye pain, blurred vision, eyestrain, and frequent blinking). The score for each category ranges from 5 to 25, with higher scores indicating greater subjective symptoms.

### 2.3. Skin Gas Analysis

**Figure 1:**
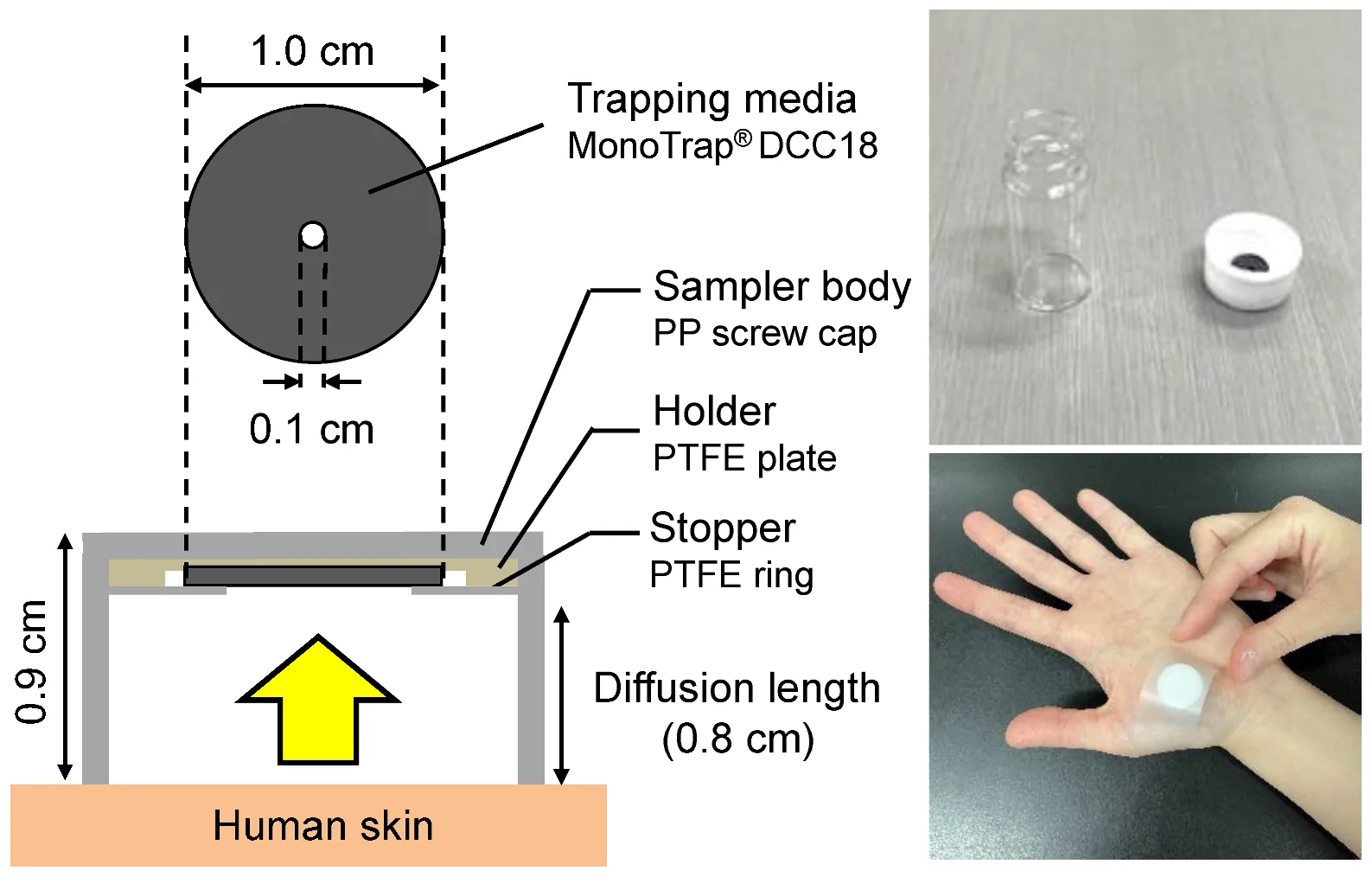
Schematic view of the passive sampler (PFS) for the collection of human skin gases emanating from skin surface. The PFS was attached to the thenar region of one palm using surgical tape.

Human skin gases were collected from the thenar region of the participants’ palms using a PFS (Figure 1). The thenar region was selected as the sampling site because the target skin gases are considered to be released directly from the blood or transported from the blood through sweat glands [17,25], and this region has a relatively high blood volume due to the presence of arteriovenous anastomoses [35].

The device structure, sampling principles, and analytical procedures of the PFS have been described in detail elsewhere [17,30,31]. Briefly, the PFS consisted of a sampler body, a trapping medium, and stoppers. The PFS was gently affixed to the skin surface of the participant’s thenar using medical tape for one hour to create a headspace. Through the open face of the sampler, skin gases emanating from the skin surface diffused toward the trapping medium within the headspace, where the gas molecules were collected. A disk-type medium composed of monolithic silica embedded with activated carbon particles (MonoTrap^®^ SG DCC 18, GL Sciences, Tokyo, Japan) was used to trap compounds such as acetaldehyde, acetic acid, acetone, propionic acid and hexanoic acid. Sampling was conducted while the participants were seated and at rest; therefore, visible moisture accumulation on the skin was not expected, and none was reported by the participants. After sampling, the PFSs were promptly shipped to Tokai University using a refrigerated courier service and stored in a freezer at 249 K until chemical analysis, which was performed according to previous studies [31].

Trapped VOCs were analysed by gas chromatography–mass spectrometry (GC–MS) after elution with 0.50 mL of carbon disulfide using ultrasonic extraction for 30 min. The analysis was performed using a gas chromatograph (model 7890B, Agilent Technologies, Santa Clara, CA, USA) coupled to a mass selective detector (JMS-Q1050GC MkII, JEOL, Tokyo, Japan). One microliter of each sample extract, blank extract, and calibration standard was injected with a split ratio of 35:1 into a DB-IMS capillary column (30 m × 0.25 mm I.D. × 0.25 µm film thickness, Agilent Technologies, Santa Clara, CA, USA) and a DB-35MS capillary column (30 m × 0.25 mm I.D. × 0.25 µm film thickness, Agilent Technologies, Santa Clara, CA, USA). The carrier gas was G1-grade helium (Taiyo Nippon Sanso, Tokyo, Japan) at a flow rate of 1.0 mL min^−1^. The injector temperature was maintained at 300 °C. The oven temperature program was as follows: held at 50 °C for 8 min, ramped at 6 °C min^−1^ to 120 °C, then ramped at 25 °C min^−1^ to 300 °C and held for 2 min. Data were acquired in real time using selected ion monitoring mode. Calibration curves were constructed using mixed standard solutions at concentrations of 0, 0.50, 1.0, and 2.5 µg mL^−1^. Excellent linearity was observed, with 0.99 of coefficients of determination (*R*^2^).

The dermal emission flux of the target skin gas i, *E*_i_ (ng cm^−2^ h^−1^) was obtained by

(1) $$E_{i}=\frac{W_{i}}{S\cdot t}$$

where *W*_i_ is a collection amount of each skin gas (ng), *S* is an effective cross-section of the trapping media (0.594 cm^2^) and *t* is a sampling duration (1.0 h). Because the dermal emission fluxes were very low, only detected values were considered, while those below the detection limit were treated as zero. All reagents were purchased from FUJIFILM Wako Pure Chemical Corporation (Osaka, Japan).

### 2.4. Two-Dimensional Mapping

As in our pilot study [22], the dermal emission fluxes of five skin gases were converted into metabolic and vasomotor indices using Equations (2) and (3), as the substantial interindividual variability in individual fluxes may limit their utility as direct indicators. The blur sensation was regarded as a subjective visual symptom that has been reported in association with psychological stress, job-related stress, and psychosocial factors [36,37]. Therefore, the “Blurriness” score was used as an indicator of perceived stress. The relationships between the two indices and the perceived blur sensation score were visualized using a two-dimensional map. A total of 441 observations from 100 participants were included in the analysis.

(2) $$Metabolic\;index=\frac{E_{acetone}}{E_{acetoaldehyde}+E_{acetic\;acid}{+E}_{acetone}}$$

(3) $$Vasomotor\;index=\frac{E_{hexanoic\;acid}}{E_{hexanoic\;acid}+E_{propionic\;acid}}$$

The observed indices were plotted in a two-dimensional coordinate system, and the corresponding “Blurriness” scores were assigned to each observation. To visualize the spatial distribution of perceived stress, a smooth response surface was estimated from the observed data using radial basis function (RBF) interpolation with a thin-plate spline kernel [38]. The interpolation was performed using the RBF Interpolator function in the SciPy library in Python 3.14.7. Interpolation was restricted to the convex hull of the observed X–Y data to avoid extrapolation beyond the range of the observations. The resulting response surface was visualized as a filled contour map, with contour lines corresponding to the selected percentile thresholds of the “Blurriness” score.

### 2.5. Trajectory Analysis

To quantify changes in the daily state represented on the two-dimensional map, the Euclidean distance between the positions of each pair of days was calculated using the data from six participants. For two observations located at (*X*_i_, *Y*_i_) and (*X*_j_, *Y*_j_), the distance was calculated as

(4) $$D_{ij}=\sqrt{{\left(X_{i}-X_{j}\;\right)}^{2}+{\left(Y_{i}-Y_{j}\right)}^{2}}$$

where *D*_ij_ represents the Euclidean distance between days i and j, and X and Y represent their respective coordinates on the two-dimensional map. Distances were calculated for all pairs of the seven consecutive days (Saturday to Friday), and the cumulative weekly movement, *D* was obtained as the sum of the *D*_ij_ between consecutive days.

### 2.6. Statistical Analysis

Statistical analyses were conducted using Microsoft Excel 365 (Microsoft Corporation, Redmond, WA, USA). Pearson’s product–moment correlation coefficients were calculated to evaluate the relationships among the scores for each category of the “Jikaku-sho Shirabe” questionnaire, the dermal emission fluxes of five skin gases, and the metabolic and vasomotor indices. Unless otherwise indicated, statistical significance was set at *p* < 0.05 and *p* < 0.01.

## 3. Results

**Figure 2:**
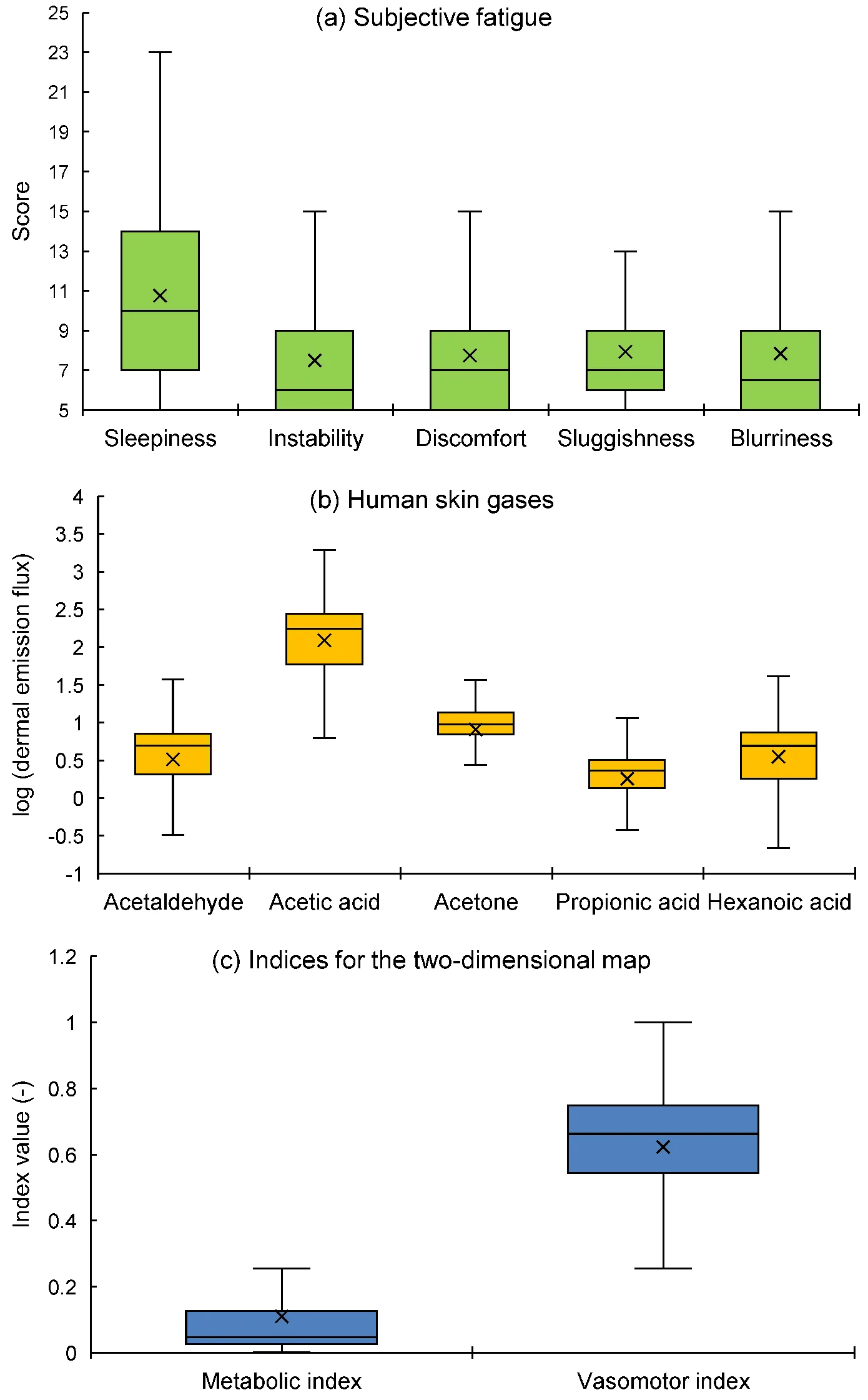
Overview of the experimental results obtained from 100 participants (*n* = 441). (**a**) Reported scores for five categories in the work-related fatigue questionnaire, “Jikaku-sho Shirabe”; (**b**) dermal emission fluxes of five human skin gases; (**c**) the metabolic and vasomotor indices. The “x” symbols in the figure indicate the arithmetic mean values.

Figure 2 shows box plots of the reported scores for the five categories of the questionnaire “Jikaku-sho Shirabe,” the dermal emission fluxes of five skin gases, and the metabolic and vasomotor indices. The “x” in the figure indicates the arithmetic mean values. In the pilot study [22], the scores for “Sleepiness” and “Blurriness” were higher than those for the other categories. In the present study, however, “Sleepiness” showed a particularly high score, and “Blurriness” score was comparable with those of other categories. The dermal emission flux of acetic acid generally tends to be higher than those of the other skin gases [17], and a similar trend was observed in the present study, with acetic acid showing the highest dermal emission flux. The metabolic and vasomotor indices were calculated from the dermal emission fluxes of the respective skin gases. Similar to the pilot study, the vasomotor index showed a larger range of variation than the metabolic index.

**Figure 3:**
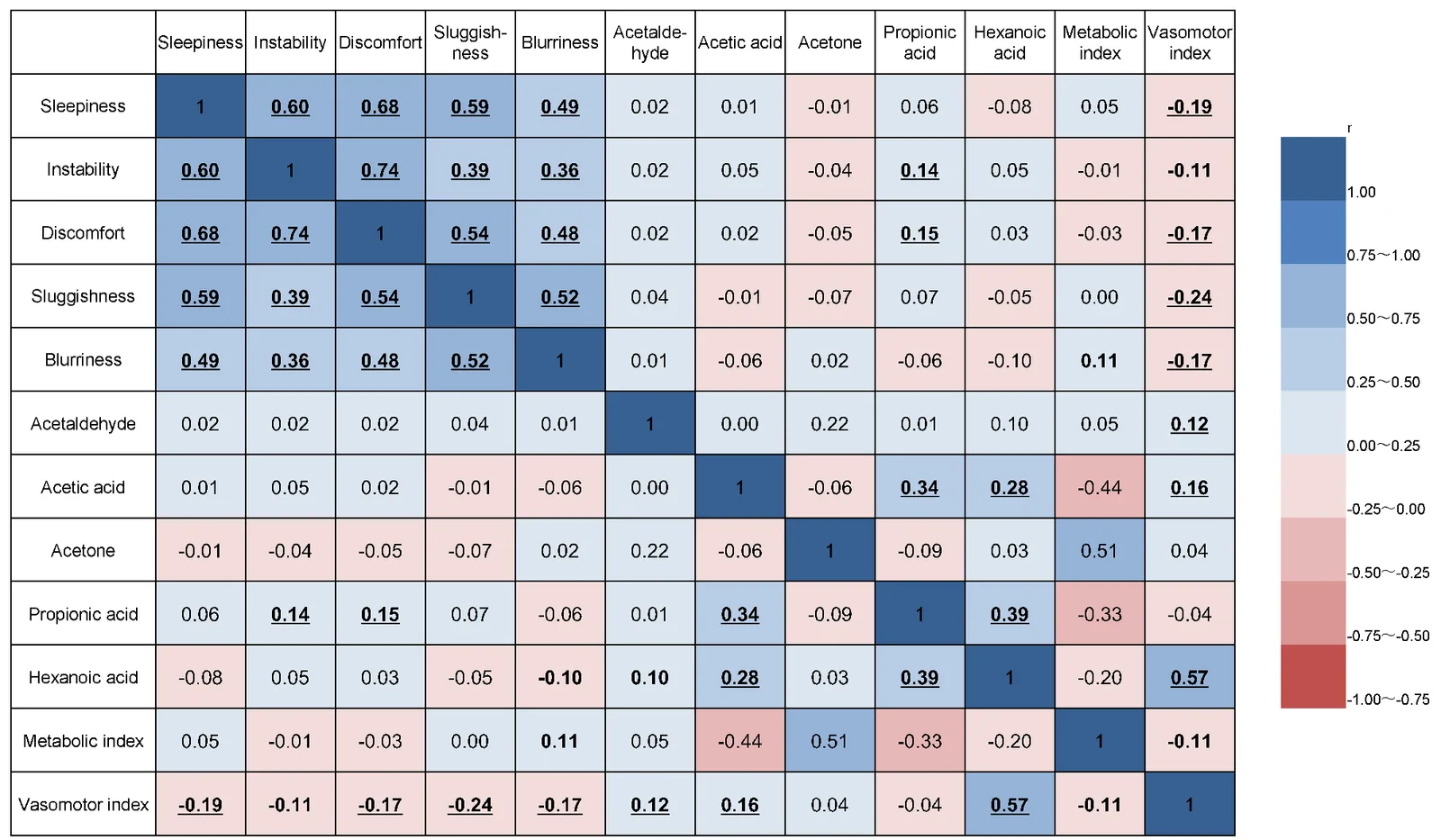
Heatmap of the correlation coefficient matrix for the reported scores of the five categories in the work-related fatigue questionnaire, “Jikaku-sho Shirabe,” the dermal emission fluxes of five human skin gases, and the metabolic and vasomotor indices. Values in bold indicate *p* < 0.05, and underlined bold values indicate *p* < 0.01.

Correlation coefficients among these parameters were calculated and presented as a heat map in Figure 3. Significant positive correlations were observed among all five scores of the questionnaire “Jikaku-sho Shirabe” (*p* < 0.01). However, the strength of the relationships differed among the categories. For “Sleepiness,” which showed the highest scores, the correlation coefficients were *r* = 0.68 with “Discomfort,” *r* = 0.60 with “Instability,” and *r* = 0.59 with “Sluggishness,” whereas the correlation with “Blurriness” was relatively lower (*r* = 0.49). “Blurriness” also showed significant correlations with all other categories, but the correlation coefficients ranged from 0.36 to 0.53, indicating relatively weak correlations.

Among the dermal emission fluxes of the skin gases, significant correlations were observed between acetic acid and propionic acid (*r* = 0.34), acetic acid and hexanoic acid (*r* = 0.28), hexanoic acid and propionic acid (*r* = 0.39), and hexanoic acid and acetaldehyde (*r* = 0.10). Acetic acid, hexanoic acid, and propionic acid are considered to be primarily emitted through the sweat gland route and may volatilize with sweating [17,25], which may explain the significant correlations among these acids. However, their production mechanisms are not necessarily the same, and only hexanoic acid showed a weak correlation with acetaldehyde.

In contrast, the relationships between individual dermal emission fluxes and the five questionnaire categories were generally weak, with significant correlations observed only between propionic acid and “Instability” and “Discomfort.” These findings suggest that individual skin gas emission fluxes alone may be insufficient to assess subjective stress-related symptoms. Therefore, as in the pilot study, we focused on the vasomotor and metabolic indices calculated using Equations (2) and (3).

The vasomotor index showed significant negative correlations with all categories of the questionnaire, consistent with the findings of the pilot study. In the pilot study, the metabolic index showed significant positive correlations with all categories except “Instability”. In the present study, however, a significant positive correlation was observed only with “Blurriness” (*r* = 0.11, *p* < 0.05). In the pilot study, a two-dimensional map was constructed using the “Blurriness” score, and the present results suggest that this framework can also be applied to the dataset of 100 participants.

**Figure 4:**
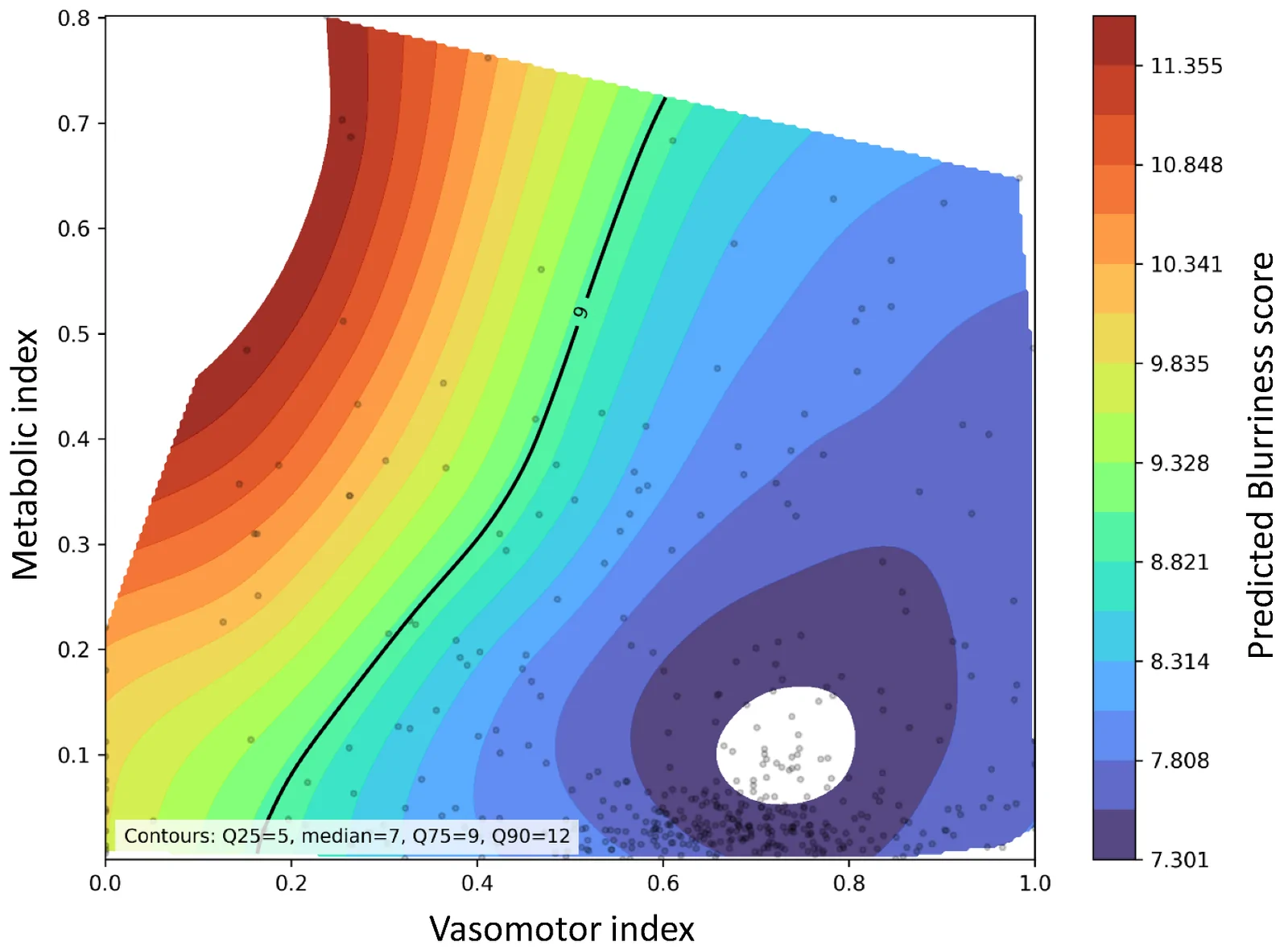
Two-dimensional coordinate map with the “Blurriness” scores represented by a continuous color scale. The black line indicates a score of 9, corresponding to the 75th percentile (Q75).

The observed vasomotor index and metabolic index were then plotted in a two-dimensional coordinate system, and the corresponding “Blurriness” scores were assigned to each observation, as shown in Figure 4. The “Blurriness” score was represented using a continuous color scale, and a black line was drawn at the score of 9, corresponding to the 75th percentile (Q75). The 75th percentile was used as a provisional threshold for visualization purposes, rather than as a cutoff based on established physiological or psychological criteria. The “Blurriness” scores were relatively low in the lower-right region of the two-dimensional map and tended to increase toward the upper-left region, supporting the findings of the pilot study. When the “Blurriness” score was regarded as a stress-related subjective symptom upon awakening, the regions corresponding to scores ≥9 and <9 were provisionally classified as high- and low-blurriness regions, respectively. Based on the position of each plot on the map derived from the skin gas measurements, differences in the stress-related subjective state among observations could therefore be visualized.

**Figure 5:**
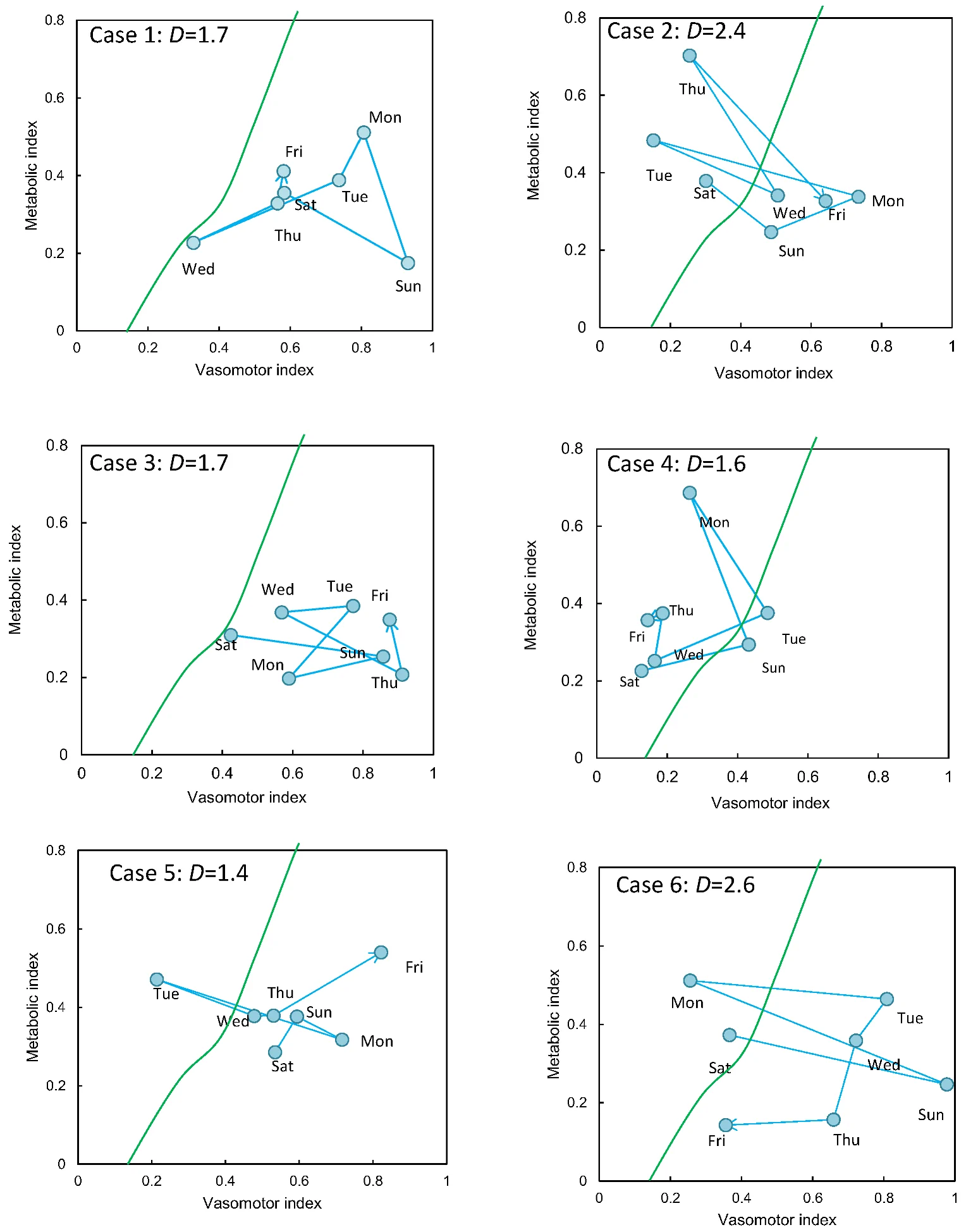
Seven-day trajectories on the two-dimensional map showing day-to-day changes in stress-related subjective states within individuals (Cases 1–6).

Because stress-related subjective states may vary from day to day, changes in the plots over seven consecutive days, from Saturday to Friday, and the cumulative weekly movement, *D*, for six participants (Cases 1–6) are shown in Figure 5. In Case 1, the plot moved toward the lower-right region from Saturday to Sunday, while the plots on weekdays tended to be located toward the upper-left relative to the Sunday plot. However, all plots remained on the lower-right side of the line corresponding to a score of 9 (Q75), suggesting that the participant remained in a relatively low stress-related subjective state throughout the week. In Case 2, the plot moved toward the lower-right region from Saturday to Sunday, whereas relatively large movements from the low-blurriness side toward the high-blurriness side were observed from Monday to Tuesday and from Wednesday to Thursday. The cumulative weekly movement was 2.4. These results indicate that the stress-related subjective state may have increased on certain days, followed by a return toward the lower-blurriness region. Case 3 showed a pattern similar to that of Case 1. In Case 4, the plots were located in the high-blurriness region except on Sunday and Tuesday. The cumulative weekly movement was relatively small (1.6), indicating that the participant remained in a relatively high stress-related subjective state for much of the week. In Case 5, the participant appeared to be in a relatively high stress-related subjective state from Tuesday to Thursday, although the cumulative movement of the plots was the smallest among the six participants. Case 6 showed the largest cumulative weekly movement among the six participants, particularly because of the large displacements from Saturday to Sunday and from Sunday to Monday.

Overall, these results indicate that the two-dimensional map based on skin gas measurements may provide a means of visualizing changes in a stress-related subjective state expressed as subjective “Blurriness” upon awakening.

## 4. Discussion

In the present study, subjective symptoms related to “Blurriness” were plotted on the two-dimensional map using vasomotor and metabolic indices to visualize an individual’s stress-related subjective state. By classifying the plotted points into low- and high-blurriness regions, it was possible to visually evaluate the stress-related subjective state at the time of awakening. Psychological stress may induce autonomic and thermoregulatory responses, including changes in thermogenesis and cutaneous vasomotor activity, which may be accompanied by changes in ocular surface homeostasis [24,39]. However, blurred vision should not be regarded as a specific indicator of stress itself, but rather as one of the subjective symptoms that may occur in association with a stress-related state. Dry eye is associated not only with dryness and foreign-body sensation but also with visual symptoms such as blurred vision, fluctuating vision, glare, and eye fatigue. Instability of the tear film and changes in the ocular surface can affect visual quality and may contribute to these symptoms [40]. Therefore, the blurred vision observed in the present study may have been affected by several factors, including psychological stress, sleep conditions, and the physiological condition of the ocular surface.

Because the subjective symptoms in the present study were assessed upon awakening, sleep conditions may be an important factor. An experimental sleep-deprivation study reported decreased tear secretion, shorter tear-film break-up time, and increased tear osmolarity in the early morning after sleep deprivation, suggesting that insufficient sleep can affect the ocular surface and tear-film homeostasis [41]. In addition, a large-scale study of approximately 100,000 Japanese individuals reported associations between unhealthy sleep status, fatigue upon awakening, and dry eye symptoms [42]. These findings suggest that morning blurred vision and other subjective symptoms may partly reflect the physiological condition during the period from the previous night through sleep to awakening.

From this perspective, the two-dimensional map obtained in the present study may be interpreted not simply as a “stress level map”, but as a method for visualizing a stress-related subjective state based on multiple subjective symptoms. Movement from the low-blurriness region to the high-blurriness region may indicate a transition toward a state with a higher level of stress-related subjective symptoms. Importantly, this classification does not mean that the individual was clinically diagnosed as being under high stress. Rather, it represents the relative position of the subjective symptom profile within the present study, because the Score = 9 line was set at the 75th percentile of the “Blurriness” scores.

The seven-day data also allowed us to examine the trajectory of the stress-related subjective state within each individual. For example, movement from the low-blurriness region to the high-blurriness or very-high-blurriness region may indicate a temporal change in the overall subjective symptom state. In contrast, clustering of points within a similar region may indicate that the subjective state remained relatively stable. Such a trajectory may provide information on within-person changes that cannot be easily identified using a single stress score at one time point.

Furthermore, the distance between coordinates on the X–Y plane can be used to quantify the magnitude of transition in the stress-related subjective state. A small distance between two consecutive days indicates that the subjective symptom states were relatively similar, whereas a larger distance indicates a greater change in the overall subjective state. Thus, the cumulative distance may provide a quantitative way to characterize individual changes in stress-related subjective states over a one-week period.

Taken together, the present findings suggest that subjective symptoms measured upon awakening may be useful for visualizing an individual’s stress-related subjective state in a two-dimensional space and for evaluating temporal changes in this state using trajectories and coordinate distances. Although blurred vision is not a specific biological marker of stress, it may be considered one of the stress-related subjective symptoms resulting from the combined effects of sleep conditions, ocular surface status, and psychological and physiological stress. An approach that integrates multiple subjective symptoms may therefore provide a useful way to describe not only a numerical stress level but also the state in which an individual is currently situated.

Several limitations should be acknowledged in the present study. Blurred vision is not specific to stress and may be influenced by sleep conditions, ocular surface status, and other factors. Moreover, the low- and high-blurriness regions were defined based on subjective symptom scores in this study and do not represent clinical criteria or diagnostic categories. Further studies incorporating objective measures of sleep, ocular conditions, autonomic activity, and psychological stress are needed to validate the proposed approach.

At present, the proposed two-dimensional map is intended to complement, rather than replace, questionnaire-based assessment. Although subjective symptom scores were used as a reference for constructing the map, skin gas measurements provide chemical information distinct from self-reported symptoms and may help characterize physiological or metabolic states associated with subjective symptoms. However, whether these measurements provide meaningful additional information beyond questionnaire-based assessment remains to be established. Furthermore, the development of sensors for rapid and simple on-site skin gas measurements may facilitate more convenient assessment in practical settings [43,44,45,46].

Skin conditions, such as dryness, lesions, and sweating, were not systematically assessed in this study. Although their effects on skin gas emissions remain unclear, future studies should investigate whether accounting for these factors could improve the comprehensiveness of the proposed two-dimensional map.

Future studies using multivariate statistical methods, such as principal component analysis (PCA) and cluster analysis, may provide further insights into the relationships between skin gas profiles and other characteristics of the study participants [16,47]. Further research is also needed to establish the practical utility of the proposed approach.

## 5. Conclusions

The present study demonstrated the potential of skin gas measurements to visualize an individual’s stress-related subjective state upon awakening. Dermal emission fluxes of five skin gases were used to derive metabolic and vasomotor indices, which were plotted on a two-dimensional map using the “Blurriness” score as a subjective reference. The spatial distribution of the “Blurriness” score was consistent with that observed in the pilot study, supporting the applicability of this framework to a larger population.

The seven-day measurements showed that trajectories on the two-dimensional map could capture day-to-day changes in the stress-related subjective state within individuals. Furthermore, the cumulative distance between consecutive coordinates provided a quantitative measure of the magnitude of these changes over one week. These findings suggest that combining skin gas-derived physiological indices with subjective symptoms may provide a useful approach for characterizing individual variations in stress-related states.

The present map does not represent a clinical stress scale, and blurred vision is not a specific marker of stress. Rather, it may reflect a subjective state influenced by multiple factors, including sleep, ocular surface condition, autonomic activity, and psychological stress. Further studies incorporating objective measures of these factors are required to validate the proposed approach and clarify the physiological basis of the observed patterns. In addition, the development of sensors for rapid and simple on-site skin gas measurements may facilitate the practical application of the proposed approach in the future.

## Data Availability

The data presented in this study are available on reasonable request from the corresponding author.

## Abbreviations

The following abbreviations are used in this manuscript:

PFS	Passive flux sampler
Q75	75th percentile
